# Fetal growth and congenital heart disease at high altitude

**DOI:** 10.1098/rstb.2024.0177

**Published:** 2025-08-21

**Authors:** Dino A. Giussani

**Affiliations:** ^1^ Department of Physiology, Development and Neuroscience, University of Cambridge, Cambridge CB2 3EL, UK

**Keywords:** high altitude, fetal growth, heart disease

## Abstract

High-altitude pregnancy lowers birth weight, and highland ancestry protects against this. Mechanisms mediating both effects are of interest, and alterations in uterine blood flow and placental metabolism have been implicated. However, this review highlights studies combining the chicken embryo model with high-altitude incubation, which reveal direct depressive effects of altitude on fetal growth and direct protective effects of highland ancestry, despite the absence of a mammalian uterine artery or placenta. Since low birth weight has been linked with high cardiovascular risk in offspring, there is also interest in the mechanisms underlying this relationship. Further work in the chicken embryo has shown that hypoxic incubation triggers congenital heart disease and programmes cardiovascular disease in the adult bird, and that highland ancestry confers protection against these effects. Therefore, these studies in the chicken embryo are important because they unveil that altitude and highland ancestry must affect prenatal growth and developmental origins of heart disease by mechanisms acting on the fetus, in addition to maternal or placental effects. The nature of these direct effects is likely epigenetic; however, they remain little studied and unidentified. Such research is paramount as it has the potential to lead to discoveries of novel fundamental biological pathways with therapeutic application of human relevance.

This article is part of the discussion meeting issue ‘Pregnancy at high altitude: the challenge of hypoxia’.

## 1. Introduction

A vast number of people live at high altitudes (>2500 m above sea level), estimated to be more than 81 million, and more than 14 million live at extreme altitudes (>3500 m) [1]. Since populations at high altitudes include women of reproductive age, this provides the largest single human group at risk of prenatal life exposure to chronic fetal hypoxia in otherwise uncomplicated pregnancy. In addition, one of the most common complications of sea-level pregnancy involves sustained reductions in fetal oxygenation. Conditions during complicated sea-level pregnancy associated with chronic fetal hypoxia include placental insufficiency, pre-eclampsia, chorioamnionitis (placental infection), gestational diabetes and maternal obesity [2,3]. Therefore, combined, gestation affected by chronic fetal hypoxia at sea level or high altitude represents a rather large component of pregnancies worldwide. Since chronic fetal hypoxia leads to fetal growth restriction and low birth weight [4], this compounds a significant clinical challenge, as both are linked with adverse health outcomes, including raised infant mortality and an increased risk of cardiovascular dysfunction in the offspring in later life [5,6]. Over the last 25 years, in the Giussani laboratory, we have been investigating the adverse side effects of chronic fetal hypoxia on fetal growth and cardiovascular development, adopting a two-pronged approach: first, by carrying out translational studies in specific human populations and, secondly, by addressing underlying mechanisms in unique animal models.

## 2. Human clinical studies in Bolivia

**Figure 1.**
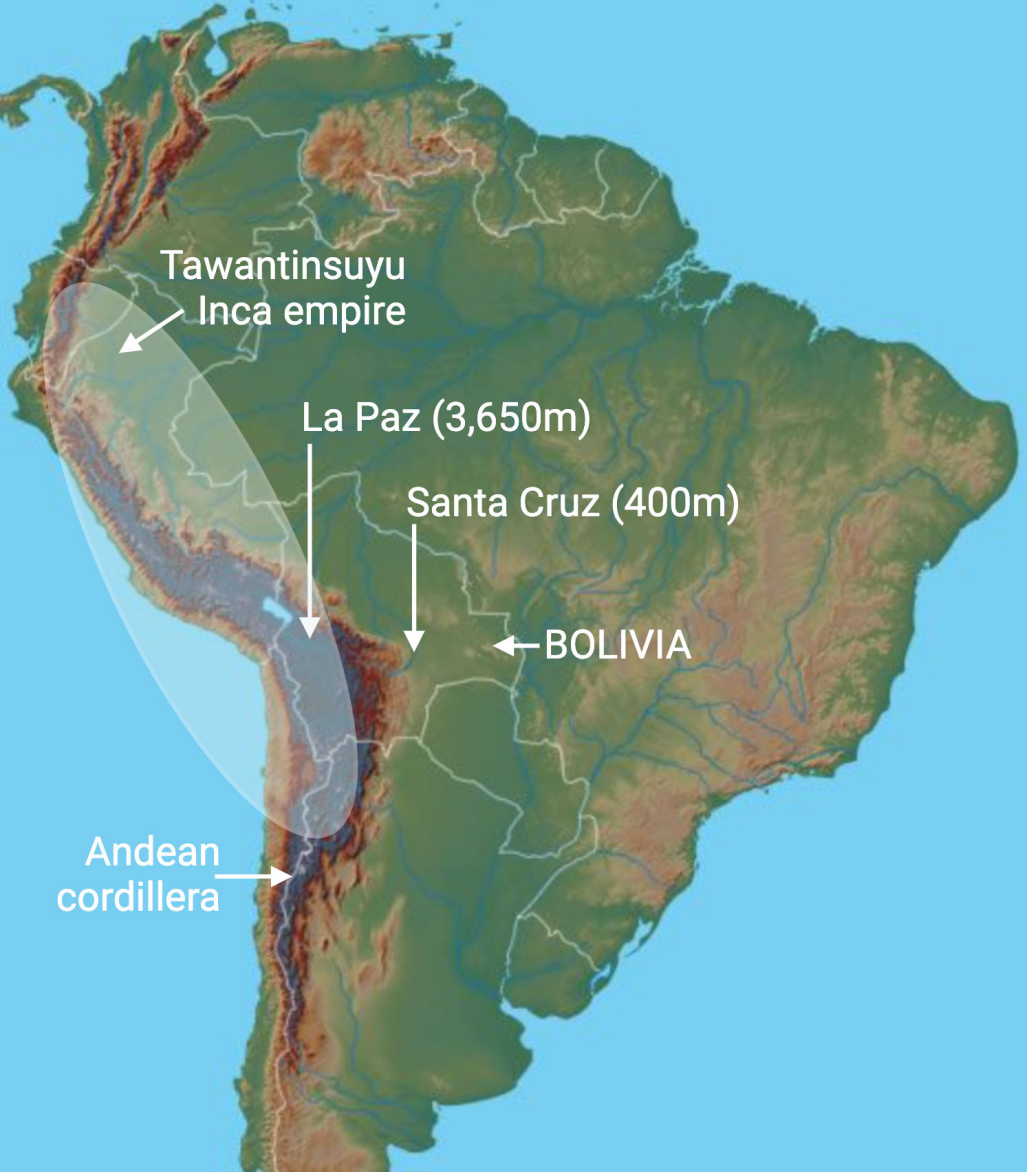
Bolivia. This country lies in the heart of South America and is split by the Andean cordillera into two areas. In the west of the country lies the highest capital city in the world, called La Paz, which stands at *ca* 4000 m. In the east of Bolivia is Santa Cruz, which stands just above sea level. Bolivia was once part of the extensive Tawantinsuyu Inca empire. Therefore, it is populated by the Aymaras, the descendants of the Incas, with a prolonged high-altitude residence ancestry, and by relative newcomers to high altitudes with a large European admixture. Created with BioRender.com.

The human clinical studies have focused on high-altitude countries in the Andes, specifically Bolivia. This country is geographically unique because it sits in the heart of South America, and it is split by the Andean cordillera into two areas (figure 1). In the west of the country lies the highest capital city in the world, called La Paz, which stands at *ca* 4000 m or 12 000 feet above sea level. The metropolitan district, formed by the combined regions of La Paz, El Alto, Achocalla, Viacha and Mecapaca, constitutes one of the most inhabited urban areas of Bolivia, with a population of *ca* 2.2 million living at extreme altitude (>3500 m). Bolivia is the only country in the world where most of its population (55%) lives at high altitude (>2500 m), and a third of its population (33%) lives at extreme altitude (>3500 m) [1]. An arterial blood sample taken from inhabitants of downtown La Paz would read approximately 60 mmHg, compared with 100 mmHg at sea level [1]. Therefore, normal life in La Paz imposes a significant challenge to physiology, even in the absence of pregnancy. In the east of Bolivia, as the country extends into the Brazilian Amazon, there are several sea-level cities. Facilitating the study design, the largest city in Bolivia is Santa Cruz, which stands at 427 m above sea level and has about 3 million inhabitants. Bolivia is also anthropologically unique because large components of its highland regions were once part of the extensive Tawantinsuyu Inca empire (figure 1). This means that, today, Bolivia is populated by inhabitants of varying highland residence ancestry. On the one hand are the Aymaras, the descendants of the Incas, with a prolonged high-altitude residence ancestry. On the other hand are the relative newcomers to high altitude, with a large European admixture.

**Figure 2.**
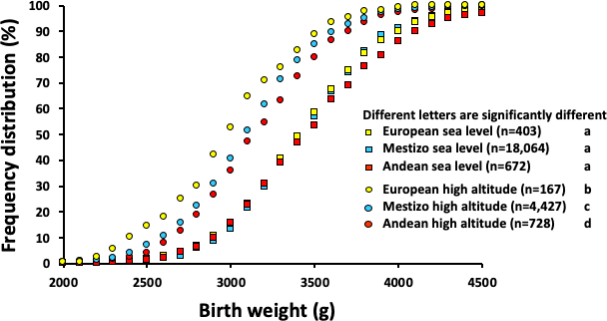
Effect of high altitude and highland ancestry on human birth weight in Bolivia. Values are the percentage cumulative frequency distribution plotted against birth weight for babies born from European (yellow circles), Mestizo (blue circles) or Andean (red circles) families following pregnancy at high altitude, or from European (yellow squares), Mestizo (blue squares) or Andean (red squares) families following pregnancy at sea level. Pregnancy at high altitude reduces birth weight, and Andean ancestry conveys graded protection against this effect. Univariate linear mixed models were used to identify the effect of high altitude and highland ancestry on birth size. Comparisons between altitude and ancestry groups were made using Student’s *t*-test for continuous variables and the *χ*^2^ test for nominal variables (SPSS, Chicago, IL). Significance was accepted when the two-tailed *p* < 0.05. Reproduced from Soria *et al*. [8], with permission.

In two seminal collaborations, several years ago, combining forces with the Instituto Boliviano de Biología de Altura, we designed a study to investigate the effect of high altitude on birth weight in healthy pregnancy and any overlaid impact of varying high-altitude residence ancestry [7,8]. In the end, close to 25 000 birth records were obtained from maternity hospitals and clinics either in La Paz or in Santa Cruz. Infant ancestry was determined retrospectively using parental surnames with a methodology validated by gene markers in this specific population [9]. As we were interested in the effects of high altitude and/or highland ancestry on birth weight in uncomplicated pregnancy, only complete records of singleton, healthy pregnancies of non-smoking mothers that reached term (>37 weeks) were assessed. When plotting the frequency distribution against birth weight and comparing with babies born from mothers of European ancestry at either La Paz or Santa Cruz, the data showed that high-altitude pregnancy induced a marked leftward shift across the whole continuum of the relationship, where average birth weight was reduced from *ca* 3.6 kg at sea level to 2.7 kg at high altitude ([6]; figure 2). The graph also shows that the prevalence of clinically defined low birth weight ([10]; <2.5 kg) in babies from European mothers at high altitude was around 15% [8]. Given that this value is between 3 and 6% of babies in the UK [10], this is an exceptionally high and significant proportion of this sub-population, considering these are normal, uncomplicated pregnancies from affluent families living at high altitude. Interestingly, babies born at high altitude from mothers with a prolonged high-altitude residence ancestry showed graded protection against the repressive effects of high-altitude pregnancy on low birth weight, and this protective effect was intermediate in Mestizo and most pronounced in Andean families ([8]; figure 2). Conversely, high-altitude residence ancestry did not have any effect on birth weight at sea level. Therefore, these studies in human pregnancy confirmed that gestational hypoxia at high altitude promotes fetal growth restriction and that multi-generational high-altitude residence ancestry confers graded protection against the effects of high-altitude hypoxia on fetal growth, being greatest in babies born from Andean mothers and intermediate in babies from Mestizo families [7,8]. Similar relationships have been reported in additional highland communities in Tibet, strengthening the argument [11].

While these data in humans were highly impacting, and reports generated from them reached the wider press [12,13], there are all sorts of limitations with this type of study. For one, as populations at high altitude are highly impoverished and have a high ethnic admixture, the partial contributions of chronic hypoxia, nutrition or genetics on fetal growth and development really remain quite unclear [7]. In addition, since the mother, placenta and fetus are all exposed to the influence of the chronic hypobaric hypoxia of pregnancy at high altitude, the partial contributions between the effects of high-altitude hypoxia on the mother, placenta and/or fetus are difficult to disentangle. Therefore, in subsequent studies, it was of interest to establish an animal model to isolate the direct effects of high-altitude hypoxia on the fetus.

## 3. Chicken embryo studies in Bolivia

In contrast to mammals, with the exception of monotremes, the chicken embryo is the ideal animal model to determine the direct effects of any environmental influence on fetal growth and development, independent of effects on the maternal and/or placental physiology, as the embryo develops in complete isolation within its own eggshell [14,15]. The model offers several other important advantages. Compared with rats and mice, which are born highly immature, cardiovascular development is near-complete pre-hatching in the chicken, and the temporal profile of these ‘prenatal’ cardiovascular developmental milestones is more similar between humans and chickens, compared with between humans and rats or mice [14,16,17]. Unlike polytocous mammals, there is no need to consider within-litter variation or the maternal metabolic investment in the pregnancy and/or the effects of lactation. Hence, there is no need for cross-fostering experimental designs to determine the effects of adverse conditions during development on longer-term outcomes in the offspring [14,15]. From an ethical perspective, there is no need to sacrifice the mother to study the fetus. Hence, the number of animals required is substantially reduced, thereby abiding strongly by the 3R principle, enshrined by the Home Office in the United Kingdom [14,15]. In addition, the chicken embryo has a short incubation period of 21 days, and eggs are highly transportable. The latter was a key necessity to be able to design a study that combined the power of the chicken embryo model with incubation at either sea level or high altitude. This allowed us, for the first time to our knowledge, to decipher the direct effects of the chronic hypoxia of high altitude, independent of effects on the mother and/or the placenta, on fetal growth and development .

**Figure 3.**
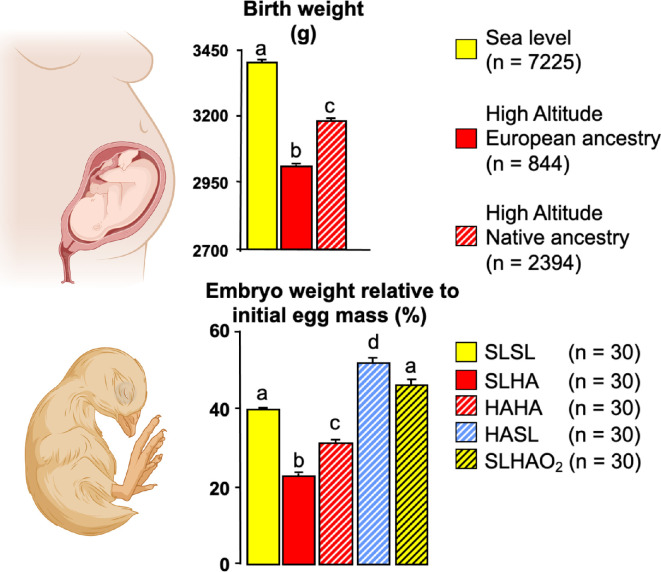
Comparative effects of high altitude and highland ancestry on growth in human babies and chicken embryos. The data in the upper graph show the mean + s.e.m. for the weight (g) at term of babies born in the sea-level city of Santa Cruz (yellow bar) and for babies of European families (red bar) or Andean families (red hatched bar) born at the high-altitude city of La Paz. The data in the lower graph show the mean + s.e.m. for the percentage embryo weight relative to the initial egg mass (at day 19 of the 21 day incubation period) for fertilized eggs laid by sea-level hens and incubated at the sea-level city of Santa Cruz (SLSL, yellow bar) or at the high-altitude city of La Paz (SLHA, red bar), or for fertilized eggs laid by multi-generational high-altitude hens and incubated at high altitude (HAHA, red hatched bar) or at sea level (HASL, blue hatched bar), or for fertilized eggs laid by sea-level hens and incubated at high altitude with oxygen supplementation (SLHAO_2_, yellow hatched bar). Different letters indicate significant (*p* < 0.05) difference (ANOVA plus Tukey test, numbers (n) in parentheses). Reproduced from Giussani *et al*. [18] and Soria *et al*. [8], with permission. Created with BioRender.com.

Therefore, in another study in Bolivia, fertilized eggs from lowland hens from Santa Cruz were incubated either at sea level in Santa Cruz or at the high-altitude city of La Paz [18]. The reciprocal experiment was also performed, where fertilized eggs were obtained from highland hens from a high-altitude farm in the El Alto region of La Paz that had produced lines of chickens for at least six generations [18]. Hence, fertilized eggs from hens with a prolonged high-altitude residence ancestry from La Paz were incubated either at high altitude in La Paz or at sea level in Santa Cruz [18]. The data showed that eggs laid by sea-level hens and incubated at high altitude were significantly growth-restricted relative to eggs laid by sea-level hens and incubated at sea level [18]. This resembled the detrimental effect of high altitude on birth weight in babies from European mothers undergoing pregnancy in La Paz relative to those in Santa Cruz ([7,8]; figure 3). When eggs laid by highland hens were incubated at high altitude, they also showed significant growth restriction relative to eggs laid by sea-level hens and incubated at sea level. However, these embryos were significantly heavier than embryos from eggs laid by sea-level hens and incubated at high altitude [18]. This resembled the protective effect on birth weight in babies from mothers of Andean ancestry relative to those of European ancestry undergoing pregnancy in La Paz ([7,8]; figure 3). Fascinatingly, eggs laid by highland hens, which usually show growth restriction when incubated at high altitude, not only recovered their growth but also grew larger when incubated back at sea level relative to eggs laid by sea-level hens and incubated at sea level ([18]; figure 3). Finally, eggs laid by sea-level hens and incubated at high altitude in La Paz with oxygen supplementation to equate sea-level partial pressures of oxygen did not show the restrictive effect of highland incubation on embryo weight ([18]; figure 3). This final group highlighted that the detrimental effect of high altitude slowing fetal growth is due to the hypoxia, rather than the hypobaria, of life at high altitude.

## 4. Mechanism of protection of highland residence ancestry on fetal growth

Naturally, the mechanisms underlying the protection of high-altitude prolonged-residence ancestry for birth weight in human babies and embryo growth in chickens are an active area of research today. However, proposed pathways remain inconclusive. Some reports suggest that, in humans, the detrimental effects of high-altitude pregnancy on birth weight may be due to alterations in utero-placental function [19]. In human pregnancy, high altitude limits uterine artery blood flow, with some studies reporting volumetric uterine artery blood flow being a third lower at 3100 m compared with 1600 m [20]. This is supported by independent studies of sheep undergoing pregnancy either at high altitude [21] or exposed to isobaric hypoxia during gestation [22]. These studies show that the increase in uterine artery blood flow with advancing gestation in control pregnancies is markedly blunted in ovine pregnancy exposed to chronic hypoxia [21,22]. This is due, in part, to an increase in constrictor reactivity coupled with a decrease in dilator reactivity in uterine arteries when gestation occurs under chronic hypoxic conditions, as has been shown in humans, sheep, guinea pigs, rats and mice [19–29]. Studies measuring uterine artery blood flow in pregnant women of varying highland residence ancestry have reported a protective effect of Andean ancestry on the effect of high altitude, reducing uterine artery blood flow. Andean women had greater uterine artery diameters, cross-sectional areas and volumetric blood flow than European women [30]. Ladakhis, like Andeans, are also protected against the repressive effect of high altitude on birth weight, particularly in families of Tibetan ancestry. Dolma *et al.* [31] also reported that in Ladakhis, uterine artery diameter at mid-pregnancy was greater in highland women giving birth to appropriate-for-gestational age compared with highland women giving birth to small-for-gestational age infants. Therefore, protection against the depressive effects of high altitude on uterine artery blood flow in families of prolonged highland residence ancestry compared with newcomers may enable the better maintenance of normal fetal growth under conditions of pregnancy at high altitude.

Another candidate mechanism mediating the effects of high-altitude pregnancy on low birth weight and protection against this effect in families of prolonged highland residence ancestry may be due to changes in placental metabolism. For instance, the maternal arterial-to-venous glucose levels are elevated in highland compared with lowland pregnancy [32]. Furthermore, increased glucose uptake has been reported in the placenta of mice exposed to hypoxia [33]. This suggests a switch in placental metabolism to favour an increased placental glucose utilization at high altitude to meet the placental ATP requirement in the face of chronic hypoxia and anaerobic glycolysis [18,32,34]. In doing so, it is possible that the placenta, while sparing fetal oxygen delivery, promotes fetal hypoglycaemia, which may in turn contribute to the reduction in fetal growth [19,32,34]. Other studies have also reported that placentas from high- compared with lowland pregnancies show greater levels of phosphocreatine and the antioxidants taurine and inositol [35]. This is consistent with reports of Tibetan women living at high altitude having placentas that are protected from labour-induced oxidative stress compared with those of other residents [36]. Studies using mouse placentas have also reported alterations in mitochondrial metabolism to best support placental growth and function, as well as fetal development, during chronic hypoxic pregnancy [37].

Interestingly, a single-nucleotide polymorphism on the *PRKAA1* gene, which encodes adenosine monophosphate-activated protein kinase (AMPK), has been linked with higher uterine artery blood flow and birth weight, as well as improved placental metabolism in multi-generational high-altitude populations [38]. The TT genotype for the rs1345778 single-nucleotide polymorphism in the α1 catalytic subunit of AMPK is associated with an increased birth weight at high altitude, and this T allele is found at higher rates in women of Andean compared with European ancestry [38]. AMPK is involved in regulating the mTOR pathway, and, in turn, the mTOR pathway is central to placental metabolic control and fetal growth [39]. Our studies in mice also support that AMPK-dependent vasodilator responses are augmented in uterine arteries isolated from mice that experienced chronic hypoxia during pregnancy [29]. Therefore, combined, data derived from human clinical studies and from mammalian animal models support that alterations in uterine blood flow and placental metabolism likely contribute to the mechanisms underlying fetal growth restriction at high altitude and the protection on birth weight conferred by prolonged high-altitude residence ancestry.

However, our chicken embryo studies in Bolivia [18] clearly highlight that protection against growth restriction by multiple generations of residence at high altitude also occurs in avian species and thereby devoid of a mammalian placenta and any influence of uterine blood flow. Therefore, these studies are important because they underscore that prolonged high-altitude residence ancestry must confer protection against fetal growth restriction by direct mechanisms acting on the fetus, in addition to effects on uterine blood flow and/or placental metabolism. These direct, and arguably more important, effects of high-altitude hypoxia on fetal growth are likely to be epigenetic. However, to date, they remain little studied and unidentified.

An interesting observation that may support epigenetic mechanisms at play is the effect of high-altitude incubation of chicken embryos on their haematocrit levels [18]. In that study, incubation at high altitude of fertilized eggs laid by either sea-level hens or multi-generational highland hens led to an increase in haematocrit compared with incubation at sea level of fertilized eggs laid by sea-level hens [18]. However, when fertilized eggs laid by highland hens were incubated at sea level, the resulting embryos not only recovered their growth but also grew heavier than sea-level controls. Furthermore, the haematocrit data revealed that this group of embryos retained an increased oxygen-carrying capacity despite incubation at sea level [18]. This suggests that embryos from multi-generational highland hens incubated at sea level had a greater oxygen content than sea-level controls, further supporting a direct role for oxygen in the control of fetal growth. The mechanism via which elevated haematocrit levels are maintained in the absence of hypoxia is unknown, but the data may reflect an epigenetic adaptive response transmitted by the mother to the oocyte prior to egg laying, predictive of fetal development under hypoxic conditions. This is similar to the well cited example of a maternal predictive adaptive response in the meadow vole [40,41]. In this species, the pre-conception photoperiodic history of the dam, rather than the perinatal thermal environment, can better predict the offspring’s coat thickness at birth [40].

## 5. Chicken embryo cardiovascular studies in Bolivia

**Figure 4.**
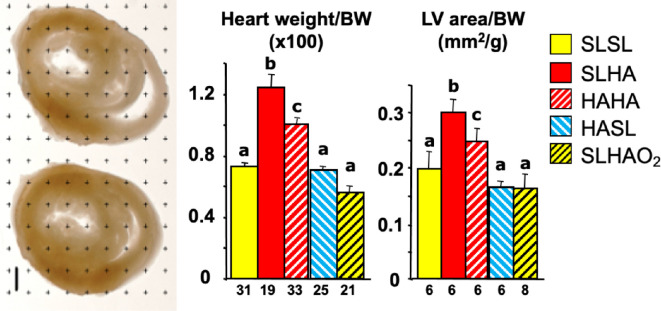
Effects of high altitude and highland ancestry on the chicken embryo heart. The data show the mean + s.e.m. for the heart weight and the left ventricular (LV) area relative to body weight (BW) at day 19 of the 21 day incubation period in chicken embryos developed from fertilized eggs laid by sea-level hens and incubated at the sea-level city of Santa Cruz (SLSL, yellow bar) or at the high-altitude city of La Paz (SLHA, red bar), or for fertilized eggs laid by multi-generational high-altitude hens and incubated at high altitude (HAHA, red hatched bar) or at sea level (HASL, blue hatched bar), or for fertilized eggs laid by sea-level hens and incubated at high altitude with oxygen supplementation (SLHAO_2_, yellow hatched bar). Different letters indicate significant (*p* < 0.05) difference (ANOVA plus Tukey test, numbers (*n*) under each histogram). Scale bar in mid-cardiac sections = 1 mm. Reproduced from Salinas *et al*. [43], with permission.

**Figure 5.**
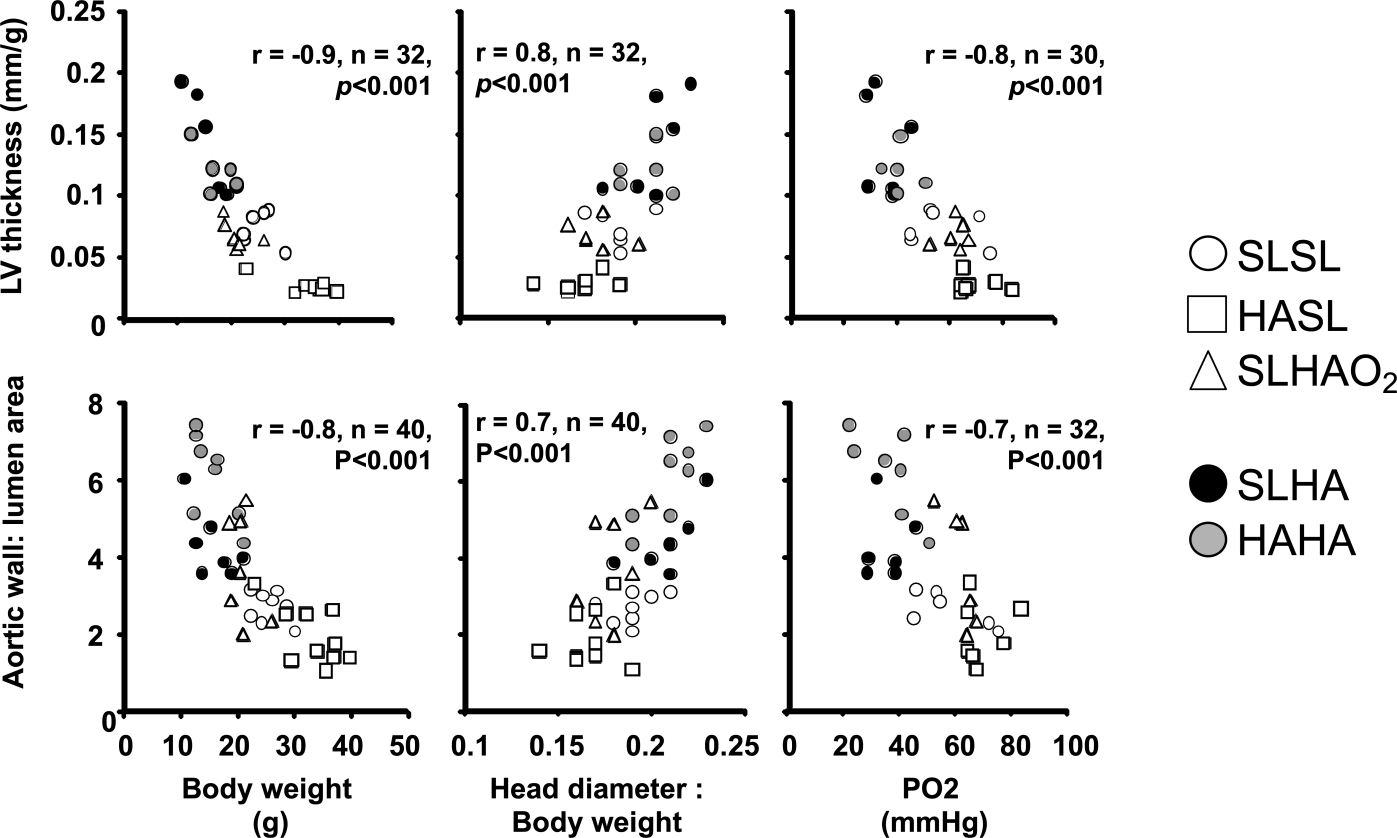
Relationship between indices of congenital heart disease, embryo growth and PO_2_ in the lowland and highland chicken embryo. The data show the relationship between the relative left ventricular thickness (top row) or the ascending aortic wall : lumen area ratio (bottom row) and body weight, the head diameter : body length ratio or the chorioallantoic artery PO_2_ at the end of the incubation period in chicken embryos developed from fertilized eggs laid by sea-level hens and incubated at sea level (SLSL) or at high altitude (SLHA), or from fertilized eggs laid by multi-generational high-altitude hens and incubated at high altitude (HAHA) or at sea level (HASL), or from fertilized eggs laid by sea-level hens and incubated at high altitude with oxygen supplementation (SLHAO_2_). Embryos incubated at sea level are shown by open symbols, and embryos incubated at high altitude are shown by filled symbols. The Pearson product–moment correlation coefficient *r*, the number of observations *n*, and the statistical significance of the relationship *p* are shown. Reproduced from Salinas *et al*. [43], with permission.

Since data derived from human clinical studies as well as experimental animal models have now established a robust relationship between fetal growth restriction and low birth weight with an increased cardiovascular risk in the offspring [5,6,42], a natural extension of our work in chicken embryos in Bolivia was to focus on cardiovascular outcomes. This analysis shows that when fertilized eggs laid by sea-level hens are incubated at high altitude, the embryos have an increase in cardiac mass and biventricular hypertrophy compared with embryos from fertilized eggs laid by sea-level hens and incubated at sea level ([43]; figure 4). Eggs laid by hens native to high altitude incubated at high altitude also show protection against this cardiac remodelling [43]. Furthermore, embryos from eggs laid by highland hens and incubated at sea level or embryos from eggs laid by sea-level hens and incubated at high altitude with oxygen supplementation also show no cardiovascular alterations [43]. When left ventricular thickness is plotted against embryo weight, a significant negative relationship is obtained (figure 5). When left ventricular thickness is plotted against the ratio of head diameter to embryo weight, a measure of asymmetric growth restriction with brain sparing [3], a significant positive relationship is obtained [43]. When left ventricular thickness is plotted against PO_2_ measured in the chorioallantoic artery (the avian homologue of the umbilical artery in mammals), a significant negative relationship is obtained [43]. Across all plots, embryos incubated at sea level or at high altitude are pushed towards the extremes of the relationships ([43]; figure 5). Combined, these data highlight the direct role of hypoxia rather than the hypobaria of high altitude in triggering embryonic origins of heart disease and protection against this in embryos from hens native to high altitude. Reports of congenital heart disease in children at high altitude are mixed. While some studies have reported an increased incidence of patent ductus arteriosus, septal defects and persistent pulmonary hypertension of the newborn [44,45], others have reported similar prevalence of structural and physiological cardiac abnormalities in high-altitude and sea-level children [46,47]. However, the cardiovascular health of children born and living at high altitude is shaped not only by the low-oxygen environment but also by population ancestry and sociocultural determinants, such as nutrition, intercurrent infection, exposure to pollutants and toxins, socioeconomic status, and access to medical care [46,47]. Interaction between these effects may explain discrepancies in the literature.

## 6. Long-term effects of hypoxic development on heart health and future work

There have now been a few studies that have reported long-term adverse effects on cardiovascular health of incubation of chicken embryos at high altitude. These studies show alterations in cardiac baroreflex sensitivity and an increased risk of pulmonary arterial hypertension in adult birds developed from eggs incubated at high altitude [48,49]. Interestingly, cardiovascular alterations are sex-dependent, and adverse effects on pulmonary pressure appear more pronounced in male rather than female birds [49]. Studies of fertilized chicken eggs incubated under isobaric hypoxic conditions also show that chronic hypoxia can act directly on the developing embryo to trigger hypertension, cardiovascular dysfunction and cardiac wall remodelling in adulthood [42,50,51]. Several other studies by independent laboratories across the world have now reported a role for chronic fetal hypoxia in programming cardiovascular dysfunction in the adult offspring of mammalian species, like mice, rats and sheep ([52,53], see [42] for review). Combined, these studies highlight a role for chronic isobaric or hypobaric hypoxia triggering long-term adverse effects on cardiovascular health in the adult offspring and that a component of this developmental programming occurs at least in part via direct effects of hypoxia on the cardiovascular system of the embryo or fetus, in addition to effects on the maternal and/or placental physiology [42,50,51]. However, systematic studies focusing on the cardiovascular health of adult birds of both sexes developed from fertilized eggs laid by hens of varying highland residence ancestry and incubated at either sea level or high altitude await investigation. Similarly, studies focusing on the cardiovascular health of babies, children or adults of both sexes born from mothers of European compared with Andean ancestry having gone through pregnancy at high altitude, incorporating more precise ancestry admixture assessments and sociocultural determinants into the statistical design, await investigation. The design of such studies is essential to gain a clearer understanding of the pathways driving the effects of the hypobaric hypoxia of high altitude on cardiovascular structure and function in the adult offspring and the mechanisms underlying any protection by prolonged high-altitude residence ancestry.

## Data Availability

This article has no additional data.
